# Serous Tubal Intraepithelial Carcinoma: An Incidental Finding at the Time of Prophylactic Bilateral Salpingo-Oophorectomy

**DOI:** 10.1155/2015/760429

**Published:** 2015-02-23

**Authors:** Monique Hiersoux Vaughan, Susan C. Modesitt, Yunchuan Mo, Elisa R. Trowbridge

**Affiliations:** ^1^Department of Obstetrics and Gynecology, University of Virginia Health System, Charlottesville, VA, USA; ^2^Thornton Gynecologic Oncology Division, Department of Obstetrics and Gynecology, University of Virginia Health System, Charlottesville, VA, USA; ^3^Department of Pathology, University of Virginia Health System, Charlottesville, VA, USA; ^4^Division of Female Pelvic Medicine and Reconstructive Surgery, Department of Obstetrics and Gynecology, University of Virginia Health System, Charlottesville, VA, USA

## Abstract

*Background.* Serous tubal intraepithelial carcinoma (STIC) is a precursor lesion for high-grade pelvic serous carcinoma. The incidence of STIC is estimated to occur in 0.6% to 6% of women who are BRCA positive or have a strong family history of breast or ovarian cancer. *Case.* A 56-year-old woman underwent robotic-assisted sacrocolpopexy, rectocele repair, and concurrent bilateral salpingo-oophorectomy for recurrent stage 3 pelvic organ prolapse and reported family history of ovarian cancer. Histopathologic examination of her left fallopian tube revealed STIC. *Conclusion.* We report this rare occurrence of STIC in a patient undergoing surgery primarily for pelvic organ prolapse and having a family history of ovarian cancer. Possible management options include observation with annual physical exam and CA-125, surgical staging, or empiric chemotherapy. However, due to the lack of consensus regarding management options, referral to a gynecologic oncologist is recommended.

## 1. Introduction

Serous tubal intraepithelial carcinoma (STIC) is a rare pathologic finding at the time of benign gynecologic surgery. It arises in the distal fimbriated end of the fallopian tube and likely represents a precursor lesion to high-grade pelvic serous carcinoma. In this case report, we describe a patient who was unexpectedly found to have STIC at the time of benign gynecologic surgery. We discuss the diagnosis, implications, management options, and prevention strategies for STIC.

## 2. Case Presentation

In March 2013, a 56-year-old G4P4 presented with recurrent stage 3 pelvic organ prolapse (apical and posterior) after total vaginal hysterectomy, transobturator TVT sling, and anterior colporrhaphy with Gynecare Prolift (Ethicon, Somerville, New Jersey) mesh in 2007. On presentation, the patient complained of a one-year history of vaginal bulge, obstructed defecation, and symptoms of urge incontinence. She was otherwise healthy with body mass index of 20 kg/m^2^ but had a 15-pack-year smoking history. On pelvic exam, she had a normal bimanual exam with no tenderness or adnexal masses. Her pelvic organ prolapse quantification system (POP–Q) exam revealed apical prolapse with the vaginal cuff located 3 cm above the hymen and posterior vaginal wall defect to 3 cm below the hymen. Additionally, the patient reported a family history of ovarian cancer in a maternal aunt in her 30s as well as possible ovarian cancer in a maternal cousin in her 30s, both still living. These family members had not undergone genetic testing. She had two healthy reproductive-age daughters. A robotic-assisted laparoscopic sacrocolpopexy, rectocele repair, and risk-reducing bilateral salpingo-oophorectomy (RRSO) were recommended. Due to her strong family history of ovarian cancer, she was offered referral to the High Risk Breast and Ovarian Cancer Clinic preoperatively, but she declined.

At the time of laparoscopy, her abdominal and pelvic surveys were normal with atrophic appearing ovaries. Her bilateral fallopian tubes and ovaries were sent for histopathologic examination. Given her family history of ovarian carcinoma, the protocol for sectioning and extensively examining the fimbria (SEE-FIM) was utilized in this case (please see a description of the SEE-FIM protocol in the discussion below). Both ovaries were also serially sectioned along the longitudinal axis and submitted in their entirety. No abnormalities were noted in either of the salpingo-oophorectomy specimens at the time of gross examination.

**Figure 1:**
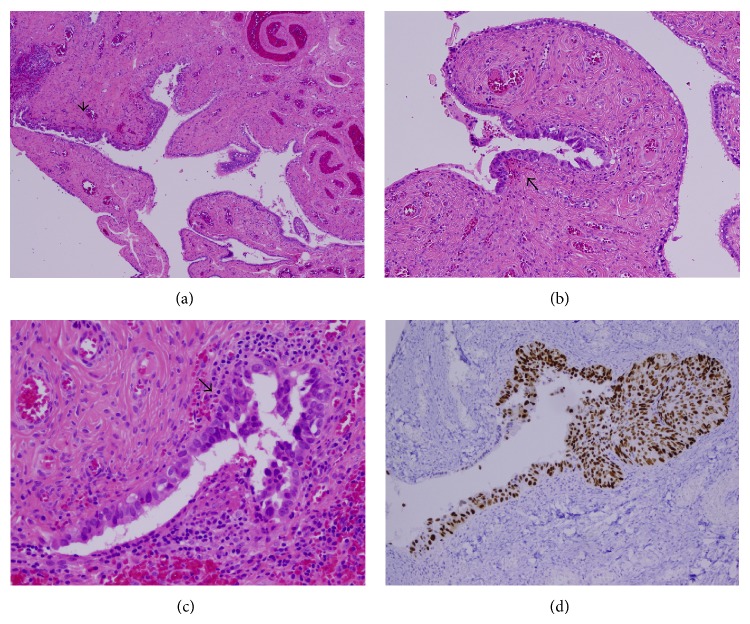
(a) H&E stain of the distal fimbriated end of the left fallopian tube. At low magnification, the area of STIC demonstrates increased epithelial thickness and nuclear stratification compared to areas of normal tubal epithelium. (b) Area of STIC with nuclear stratification and moderate variability in size and shape. Also note the absence of ciliated cells within the lesional area. (c) At high magnification, nuclear atypia is clearly visible, including hyperchromatism, prominent nucleoli, loss of polarity, and increased mitotic figures. (d) Strong and diffuse nuclear TP53 staining of STIC.

She had an uneventful postoperative course and was discharged home on postoperative day number 2. Histopathologic examination of the specimen returned with serous tubal intraepithelial carcinoma of the fimbriated portion of the left fallopian tube. The left ovary along with the right fallopian tube and right ovary revealed no pathologic abnormality.  Figure 1 depicts the histologic findings of STIC, including nuclear atypia, loss of polarity, increased mitoses, and positive p53 staining.

These findings were discussed with the patient and she was referred for gynecologic oncology consultation, where management options were discussed. These included observation with close follow-up, repeated surgery with biopsies and staging, or empiric chemotherapy. She chose to proceed with observation and is followed up every 6 months with pelvic exam and CA-125. Her last CA-125 was normal at 5 u/mL. At her initial visit with gynecologic oncology, the patient was very fearful of genetic testing due to concerns that it was “experimental.” She subsequently met with a genetic counselor but continues to decline genetic testing.

## 3. Discussion

The incidence of STIC has primarily been studied in patients with known BRCA mutations or a strong family history of breast or ovarian cancer and is estimated to be in the range of 0.6% to 6% [1–3]. Wethington et al. [2] found the incidence of STIC in women undergoing risk-reducing salpingo-oophorectomy (RRSO) at a single institution to be 2%. All of the patients in that study had a known BRCA mutation or high-risk personal or family history. Rabban et al. [4] performed a pathologic evaluation of the fallopian tubes of women at low risk for hereditary breast and ovarian cancer undergoing benign gynecologic surgery. STIC lesions were identified in 4 out of 522 cases (0.76%). STIC is a pathologic finding of unclear clinical significance, but it appears to be a precursor lesion for high-grade pelvic (tubal, ovarian, or primary peritoneal) serous carcinoma arising in the distal fimbriated end of the fallopian tube [5, 6]. Evidence supporting this theory includes the following: approximately 10–15% of fallopian tube STIC cases are detected in women undergoing RRSO for BRCA 1 and 2 mutations [1] and STIC is detected in approximately 50–60% of cases of sporadic pelvic high-grade serous carcinoma [7]. Additionally, patients with concurrent pelvic serous tumors and STIC have been shown to have identical TP53 mutations in both the metastatic tumor and the fallopian tube precursor, suggesting clonality [8]. Finally, there have been compelling studies in recent years demonstrating that many high-grade serous “ovarian” cancers actually originate in the fallopian tube mucosa. Evidence supporting this theory came originally from RRSOs in BRCA mutation carriers, where many occult ovarian cancers were found to have tubal involvement. Given this new data, the fallopian tubes of high-risk women are now more closely scrutinized (see below regarding the SEE-FIM protocol) [9, 10].

There are currently no standardized criteria for diagnosis of STIC lesions, as the histopathologic spectrum is very wide and no two STICs appear exactly the same. However, STIC is traditionally diagnosed with a combination of histopathologic and morphologic evaluation with immunohistochemical staining. Histopathologic features of STIC include a variable combination of epithelial stratification (including loss of cellular polarity and exfoliation of cells into the lumen), intraepithelial fracture lines, irregular luminal surface, pleomorphism, loss of ciliation, irregular chromatin pattern, nuclear rounding, nuclear molding, increased nuclear-to-cytoplasmic ratio, mitotic figures, and apoptotic bodies [11]. Morphologic features evident from low power include architectural disarray and increased nuclear stratification of the tubal epithelium without evidence of stromal invasion. High-power examination demonstrates individual cells with enlarged nuclei resulting in an increased nuclear to cytoplasmic ratio, prominent nucleoli, loss of polarity, and lack of ciliated cells. Immunohistochemical staining usually is positive for TP53 and Ki-67 [2, 4]. TP53 is a tumor suppressor gene that is typically mutated in STIC and pelvic serous tumors. The overexpressed, nonfunctional P53 protein accumulates, leading to strong, nuclear reactivity in a confluent distribution. Conversely, Ki-67 is a protein marker of cellular proliferation and is typically overexpressed in STIC and pelvic serous tumors. Finally, immunohistochemical staining for STIC may reveal overexpression of phosphorylated histone H2AX (*γ*-H2AX), which is an early cellular response to DNA double stand breaks, as well as overexpression of Bcl-2 (an antiapoptotic protein) that may indicate a change to a secretory phenotype, with complete loss of ciliated cells in the fallopian tube [12].

Given this patient's reported family history, the SEE-FIM protocol was utilized for histologic examination of her fallopian tubes. Initially implemented in patients with documented BRCA mutations, this protocol involves close examination and submission of the entire fallopian tube for histologic evaluation. The proximal fallopian tube is serially cross-sectioned at 2.0-3.0 mm intervals, and the distal fimbriated end is longitudinally sectioned for maximal exposure of the fimbrial epithelium [13].

Given the rarity of an STIC diagnosis, there are limited data on clinical outcomes or management strategies. Wethington et al. [2] retrospectively evaluated 593 high-risk women who underwent risk-reducing salpingo-oophorectomy and concomitant peritoneal washings. Twelve patients (2%) were diagnosed with STIC, with one patient having positive peritoneal cytology. Subsequently, seven of those patients had completion surgical staging and none was found to have any further malignant pathology. Additionally, those patients were followed up with individualized combinations of CA-125 testing, imaging, and pelvic exam for roughly 28 months, and no recurrences were diagnosed. Although this study had a small sample size, the results suggest that the yield of surgical staging is low, and short-term clinical outcomes are favorable [2]. There is no current consensus among gynecologic oncologists regarding appropriate management for incidental findings of STIC. Proposed management strategies of STIC include close surveillance, surgical staging, or empiric adjuvant chemotherapy. Close surveillance could be performed with annual review of systems, pelvic exam, CA-125, and/or imaging. As demonstrated by Wethington et al. [2], the value of surgical staging may be low, but performing a pelvic washing at the time of RRSO may be beneficial in evaluating for spread of disease. As positive peritoneal washings indicate the presence of circulating premalignant cells in the peritoneal cavity, it may be reasonable to offer patients with positive peritoneal cytology empiric chemotherapy [2]. For women with only STIC in the absence of positive washings or evidence of malignant spread, empiric adjuvant chemotherapy similar to what would be recommended by National Comprehensive Cancer Center Network (NCCN) guidelines for stage I ovarian or fallopian tube cancer (3–6 cycles of paclitaxel/carboplatin chemotherapy) could be given [14]. However, given the possibility of adverse effects, a risk-benefit ratio should be performed between the physician and patient prior to initiating chemotherapy. More studies are needed assessing effective management strategies but will be limited by low numbers.

It is also important to describe prevention strategies for STIC and invasive pelvic high-grade serous carcinoma. Given an estimated 80–90% of BRCA-related “ovarian” cancers originating in the fallopian tube, ACOG and the NCCN recommend that all women known to have a BRCA 1 or 2 mutation (or other hereditary cancers associated with ovarian cancer, like Lynch syndrome) undergo prophylactic RRSO by the age of 40 or at completion of childbearing, as there are extensive limitations to current ovarian cancer screening strategies [14, 15]. Of note, approximately 80% of ovarian cancer is not hereditary and thus there has been a strong movement toward the consideration of bilateral salpingectomy in all patients undergoing benign gynecologic surgery, whether BRCA status is known or not, in an attempt to further reduce ovarian cancer risk for all women. Other risk reducing measures include the use of oral contraceptive pills, which can reduce ovarian cancer risk by 50% if taken for 5 years [14].

Particularly challenging in this case was the patient's refusal to undergo genetic testing in light of her significant family history of ovarian cancer and personal STIC history. The knowledge of her BRCA status could affect which screening options she is offered for breast cancer surveillance and could also impact her family members. If she were positive for a BRCA germline mutation, immediate BRCA testing could be implemented in her family members to either confirm their high-risk status or clear them of risk. If her daughters were found to have a mutation, it would be imperative to discuss heightened cancer screening for breast/ovarian cancer as well as both surgical and nonsurgical prevention measures. For now, she continues to be evaluated every six months with pelvic exam and CA-125 level.

All gynecologic surgeons, including those practicing female pelvic medicine and reconstructive surgery, should have an understanding of the risk of fallopian tube and ovarian cancers. Surgeons should consider removal of fallopian tubes on all patients undergoing surgery for pelvic organ prolapse, as approximately 80% of “ovarian” cancers arise in women without known familial etiology. However, given fallopian tubes and ovaries that can be technically challenging to remove during vaginal surgery, urogynecologists should at least consider bilateral salpingectomy or risk-reducing BSO in patients who have a strong family history of breast/ovarian cancer or those that are known BRCA mutation carrier. In patients undergoing robotic surgery, where it is less technically challenging to remove fallopian tubes, consideration should be made for bilateral salpingectomy on all patients.

## References

[1] Finch A., Shaw P., Rosen B., Murphy J., Narod S. A., Colgan T. J. (2006). Clinical and pathologic findings of prophylactic salpingo-oophorectomies in 159 BRCA1 and BRCA2 carriers. *Gynecologic Oncology*.

[2] Wethington S. L., Park K. J., Soslow R. A. (2013). Clinical outcome of isolated Serous tubal intraepithelial carcinomas (STIC). *International Journal of Gynecological Cancer*.

[3] Manchanda R., Abdelraheim A., Johnson M. (2011). Outcome of risk-reducing salpingo-oophorectomy in BRCA carriers and women of unknown mutation status. *BJOG: An International Journal of Obstetrics and Gynaecology*.

[4] Rabban J. T., Garg K., Crawford B., Chen L.-M., Zaloudek C. J. (2014). Early detection of high-grade tubal serous carcinoma in women at low risk for hereditary breast and ovarian cancer syndrome by systematic examination of fallopian tubes incidentally removed during benign surgery. *American Journal of Surgical Pathology*.

[5] Ishikawa H., Kiyokawa T., Utsuno E., Matsushita K., Nomura F., Shozu M. (2014). Serous tubal intraepithelial carcinoma in a Japanese woman with a deleterious BRCA1 mutation. *Japanese Journal of Clinical Oncology*.

[6] Nasser S., Arsenic R., Lohneis P., Kosian P., Sehouli J. (2014). A case of primary peritoneal carcinoma: evidence for a precursor in the fallopian tube. *Anticancer Research*.

[7] Callahan M. J., Crum C. P., Medeiros F. (2007). Primary fallopian tube malignancies in *BRCA*-positive women undergoing surgery for ovarian cancer risk reduction. *Journal of Clinical Oncology*.

[8] Kindelberger D. W., Lee Y., Miron A. (2007). Intraepithelial carcinoma of the fimbria and pelvic serous carcinoma: evidence for a causal relationship. *The American Journal of Surgical Pathology*.

[9] Modesitt S. C. (2013). What is new in gynecologic oncology? Thought-provoking articles from the past year. *Obstetrics and Gynecology*.

[10] Crum C. P., McKeon F. D., Xian W. (2012). The oviduct and ovarian cancer: causality, clinical implications, and ‘targeted prevention’. *Clinical Obstetrics and Gynecology*.

[11] Vang R., Shih I.-M., Kurman R. J. (2013). Fallopian tube precursors of ovarian low- and high-grade serous neoplasms. *Histopathology*.

[12] Chene G., Cayre A., Raoelfils I., Lagarde N., Dauplat J., Penault-Llorca F. (2014). Morphological and immunohistochemical pattern of tubo-ovarian dysplasia and serous tubal intraepithelial carcinoma. *European Journal of Obstetrics & Gynecology and Reproductive Biology*.

[13] Medeiros F., Muto M. G., Lee Y. (2006). The tubal fimbria is a preferred site for early adenocarcinoma in women with familial ovarian cancer syndrome. *The American Journal of Surgical Pathology*.

[14] National Comprehensive Cancer Network (2014). *Genetic/Familial High-Risk Assessment: Breast and Ovarian (Version 1.2014)*.

[15] American College of Obstetricians and Gynecologists (2009). ACOG Practice Bulletin No. 103: hereditary breast and ovarian cancer syndrome. *Obstetrics & Gynecology*.

